# The molecular mechanisms on glomangiopericytoma invasion

**DOI:** 10.1186/1750-1172-8-152

**Published:** 2013-09-29

**Authors:** Qiang Sun, Chunye Zhang, Wantao Chen, Yue He

**Affiliations:** 1 Department of Stomatology, The First Affiliated Hospital of Zhengzhou University, No.1, East Jian she Road, Zhengzhou, Henan Province 450052, China; 2 Department of Oral and Maxillofacial-Head and Neck Oncology, Ninth People’s Hospital, Shanghai Jiao Tong University School of Medicine, 639 Zhizaoju Road, Shanghai 200011, China; 3 Department of Oral Pathology, Ninth People’s Hospital, Shanghai Jiao Tong University School of Medicine, Shanghai 200011, China

**Keywords:** Glomangiopericytoma, Skull base area, Sinonasal hemangioperictyoma

## Abstract

**Purpose:**

To observed the imaging and pathological features of the glomangiopericytoma.

**Experimental design:**

In this paper we report a typical case of glomangiopericytoma arising in the skull base area and summarize the clinical manifestations, imaging and pathological features of such diseases.

**Results:**

Immunohistochemical staining confirmed the tumor cells were strongly positive to Vim, SMA, MSA and negative to CD31, CD34. Partial cells were positive to FVIII. The imaging can’t confirm the diagnosis but indicate the the tumor has intact envelope.The cells in the tumor envelope is positive to Vim and negative SMA and FVIII. These findings were compatible with glomangiopericytoma and the cells in the tumor envelope is not glomangiopericytoma cells.

**Conclusion:**

In view of the clinical and pathological features of the glomangiopericytoma, we believe that the surgery is the best treatment so far and the tumor can be resected completely. The above results can be preliminary reason to explain the low recurrence of such diseases.

## Introduction

Glomangiopericytoma is belonged to a spectrum of lesions which includes myofibromatosis, myofibroma, infantile haemangiopericytoma and myopericytoma. Glomangiopericytoma was first reported as hemangiopericytoma [1], but this definition has been questioned [2,3]. In recent years, the concept of hemangiopericytoma has been evolving to myopericytoma because the spindle cells show myoid differentiation with positivity for smooth muscle actin [4]. Glomangiopericytoma have characteristics which have a component of cells with golmus-type features including cuboidal shape, distinct cell borders, clear to eosinophilic cytoplasm and central round nuclei. The etiology is not clear although past trauma, hypertension, pregnancy and use of corticosteroids may be involved [5].

In the last 20 years, only four cases of patients with oral and maxillofacial tumor in our hospital were diagnosed as hemangiopericytoma and two cases of which were myofibromatosis, one case was glomangiopericytoma, the other can’t be classified clearly(Table 1). In this paper we report a typical case of glomangiopericytoma arising in the right skull base area and summarize the clinical manifestations, imaging and pathological features of such diseases.

**Table 1:** Clinical information of patients with hemangiopericytoma

**Disease site**	**Age, y**	**Sex (Female, Male)**	**Pathology**	**IHC**	**Risk factors (trauma, hypertension and steroid useare)**	**Recurrence**
Bucca	65	Female	hemangiopericytoma	Vim(+),SMA(+), CD31(+), CD34(+)	No	No
Skull base area	55	Female	glomangiopericytoma	Vim(+),SMA(+), MSA(+), CD31(-), CD34(-)	No	No
Mandible gum	39	Male	myopericytoma	Vim(+),SMA(+), MSA(+), CD31(-), CD34(-), Des(-)	No	No
*Dorsum of tongue*	58	Female	myopericytoma	Vim(+),SMA(+), CD34(-), Des(-)	No	No

## Method

This study was approved by Institutional Ethic Committee Office of Shanghai Ninth People’s Hospital (reference number: 2013(88)). Written informed consent was obtained from the patient for the publication of this article and accompanying images.

## Result

### Case report

A 55-year-old woman presented with a 2-month history of right parotid area tumor and intermittency pain, and the patient feel foreign body sensation in right pharyngeal portion. Type-B ultrasonic revealed a solid tumor possibly arising from the deep part of parotid gland. The skin of the parotid gland was free of the tumor. We performed the surgery within a month after the first visit to our department, the patient claimed that the foreign body sensation become serious and the tumor size increased gradually. The border of the tumor is evident and the tumor has intact envelope but which bled very easily, so we did not perform biopsy. With these clinical characteristics and radiographic findings, we supposed the tumor to be benign hypervascular tumor, such as neurinoma, although the possibility of metastatic cancer was not excluded. Considering the location, the tumor occupy the overall right skull base area and which oppress the interal carotid artery, we concluded that the tumor was resectable through submandibular incision and the angle of mandible was cut off. The tumor don’t have obviously stem, and the tumor was removed.

### Imaging characteristics

**Figure 1:**
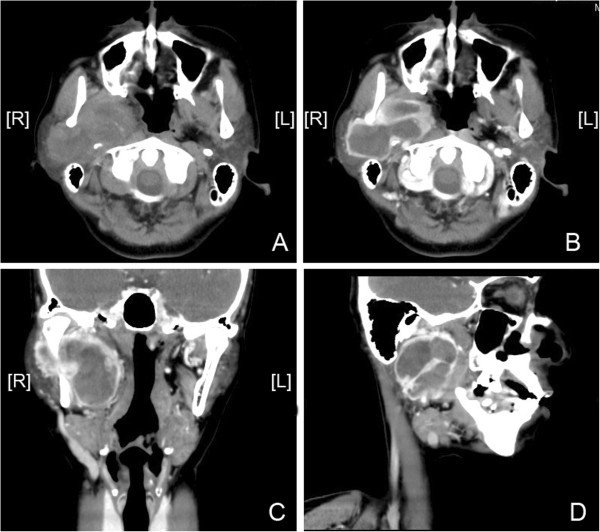
**CT images. A** is CT plain scan. **B**, **C**, **D** is enhanced CT scan. **B** is plain scan image, **C** is coronal scan image and **D** is sagittal scan image.

**Figure 2:**
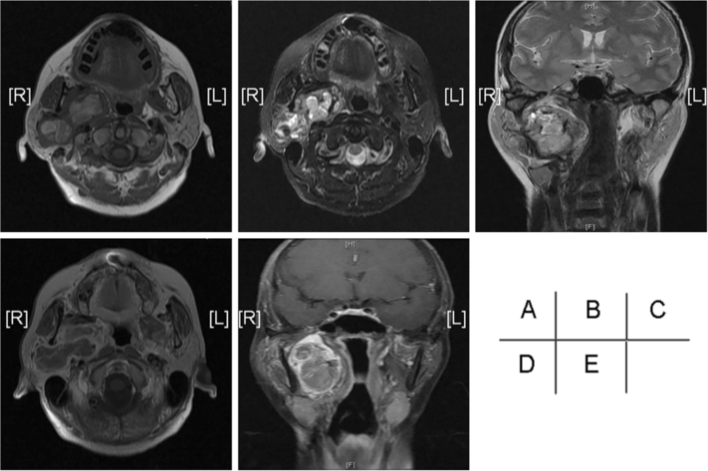
**MR images. A**, **B**, **C** is MRI plain scan. **A** is transverse TIWI, **B** is the axial pressure grease T2WI and **C** is coronal T2W1. Figure **D** and **E** is enhanced MRI, **D** is transverse T1W1 and **E** is coronal fat suppression T1W1.

Enhancement CT showed a lesion consisting of about 6.2 cm × 4.9 cm × 2.3 cm soft tissue density in the the deep part of parotid gland and the lesions showed marked heterogeneous enhancement. The right jugular vein and lateral pharyngeal wall was oppressed and shifted, the deep surface of mandibular ramus was oppressed and attenuated (Figure 1). The CT diagnosis: tumor in the parotid deep leaves which may be diagnosed as neuroma or Adenoid Cystic Carcinoma and the MRI examination is recommended. Magnetic Resonance shows a solid tumor with clear boundary and showed a lobulated tumor, magnetic resonance T1W1 performance high-mix signal (Figure 2A). After T2W1 and fat suppression show level of mixed signal (Figure 2B, C) and Contrast-Enhanced Magnetic Resonance showed inhomogeneous enhancement in the tumor and the tumor envelope is rich in blood (Figure 2D, E). The diagnose of MRI was tumor arising from parotid deep leaves and can’t confirm the diagnosis.

### Pathological features

**Figure 3:**
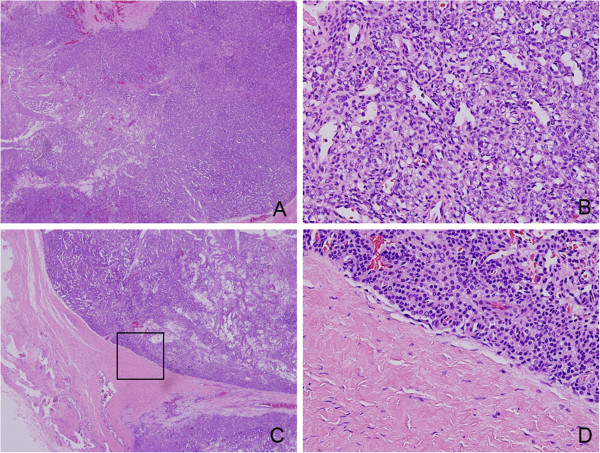
**Hematoxylin and eosin (HE) images. A** (40×) and **B** (400×) is HE staining of the operative specimen center section. **C** (40×) and **D** (400×) is HE staining of the operative specimen edge.

**Figure 4:**
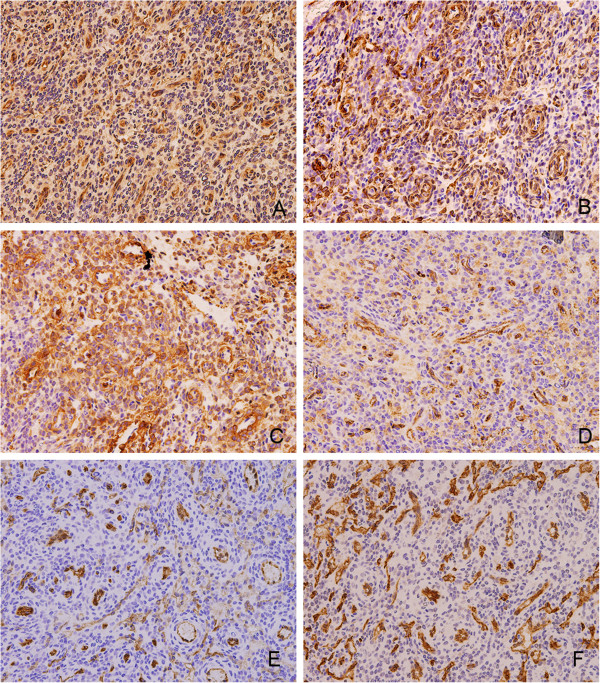
**Immunohistochemistry images of the tumor ceter. A** (400×) shows that tumor cells are positive to Vim. **B** (400×) shows that tumor cells are positive to SMA. **C** (400×) shows that tumor cells are positive to MSA. **D** (400×) shows that partial tumor cells are positive to FVIII. **E** (400×) shows that partial tumor cells are negative to CD31. **F** (400×) shows that partial tumor cells are negative to CD34.

**Figure 5:**
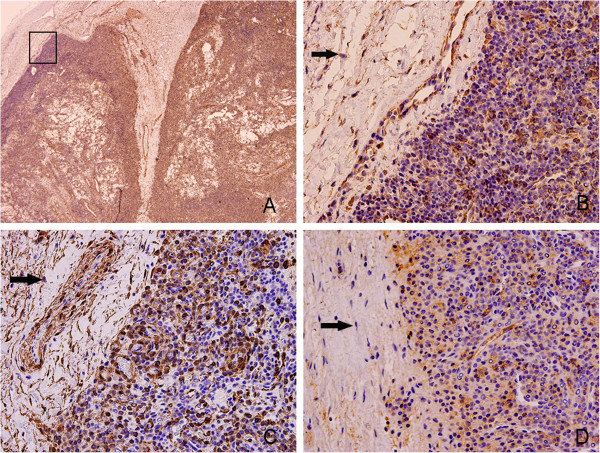
**Immunohistochemistry images of the tumor edge. A** (40×) shows the overall situation of the tumor margin. **B** (400×) shows that the cells within the tumor envelope (showed by the arrow) are negative to Vim. **C** (400×) shows that the cells within the tumor envelope (showed by the arrow) are negative to SMA. **D** (400×) shows that the cells within the tumor envelope (showed by the arrow) are negative to FVIII.

Histological examination of the tumor revealed the tumor was rich in glomus-like round cells. Hematoxylin and eosin (H&E) staining of the operative specimen showed a variable numbers of vascular channels and perivascular concentric cellular growth (Figure 3A). Glomus-like round cells were arranged around the vessels. The tumor has a rich vasculature ranging from slit-like sinusoidal spaces to dilated thin-walled vessels. Nuclear pleomorphism is absent and mitotic figures are low (Figure 3B) and we can see the tumor has intact envelope (Figure 3C, D). This constellation of findings is characteristic of glomangiopericytoma (GPC). Immunohistochemical staining confirmed the tumor cells were strongly positive to Vim, SMA, MSA (Figure 4A, B, C) and negative to CD31, CD34 (Figure 4E, F). Partial cells were positive to FVIII (Figure 4D). These findings were compatible with glomangiopericytoma. The cells in the tumor envelope is positive to Vim and negative SMA and FVIII (Figure 5), the above results suggest that the cells in the tumor envelope is not glomangiopericytoma cells.

## Discussion

Glomangiopericytoma has been diagnosed as hemangiopericytoma in the past years [6]. Haemangiopericytoma were defined as a group of tumors that develop in the head and neck region [7], and the WHO unified similar concepts into the category of glomangiopericytoma in 2005. They have a better prognosis despite their similarity to the conventional tumor, and all the four patients above mentioned have not recurrence. They often have histological features that look different from haemangiopericytomas if the tumor cells closely resemble glomus cells and these tumor cells are characterized by round, punched-out central nuclei and pale eosinophilic cytoplasm. The term glomangiopericytoma or sinonasal-type haemangiopericytoma is preferred to describe them. Many cases which mainly arising in the nasal cavity, paranasal sinuses and other parts have been reported in journals [6,8,9], but there has been no previous report of glomangiopericytoma in the skull base area. We present a case of glomangiopericytoma of the skull base area. It is worthy to note because it is rare to find such tumors in that region. It is difficult to differentiate the diagnosis from histologically dissimilar lesions that include glomangioma, myopericytoma and angioleiomyoma and differential diagnosis with pleomorphic adenoma of parotid gland, neurinoma, Adenoid Cystic Carcinoma and tumor derived from the meninges et al. In our case, the glomus like cells were arranged in a concentric perivascular array with vimentin, SMA, MSA, CD99, S-100, Bcl-2 positivity and CD31, CD34, CK9, EMA, HMB45, Melan A negativity, which are the histological characteristics of glomangiopericytoma. There have been more than 100 cases of hemangiopericytoma-like tumors have been reported in the nasal cavity and perinasal sinuses [9], The pathogenesis of glomangiopericytoma is unclear. Although trauma, hypertension and long term steroid use are were specuted to be possible causes of the tumor [5], the patient denied such histories. The etiology for this patient’s tumor is unclear. Further research into the pathogenesis and prognosis of glomangiopericytoma is needed.

Glomangiopericytoma is considered to be low malignant tumors which have low propensity to metastasize, a recurrence rate of 7% to 40%, and a 5-year survival of 88% or higher [10,11] and the patients undergoing surgery in our part all have no recurrence. The above phenomenon can get a preliminary explanation. As the HE images show that the tumor has intact envelope and there is clear boundaries between the tumor and surrounding normal tissues (Figure 1C,D). The important thing is that the cells in envelope is not tumor cells and this can be proved by the immunohistochemical staining (Figure 5B, D, E).

In summary, in view of the clinical and pathological features of the glomangiopericytoma, we believe that the surgery is the best treatment so far and the tumor can be resected completely. Of crouse, the large sample search is necessary.

## Competing interest

The authors declare that they have no competing interests.

## Grant Support

This work was supported by grants of the National Natural Science Foundation of China (NSFC30600714, 30973341), and ShuGuang project (10SG19) supported by Shanghai Municipal Education Commission and Shanghai Education Development Foundation, Specialized Research Fund for the Doctoral Program of Colleges and Universities (20110073110078) and Doctoral Innovation Foundation Award (BXJ201132) from Shanghai Jiao Tong University School of Medicine.

## Authors’ contributions

QS collected Clinical data and drafted the manuscript, CZ carried out the molecular studies and pathological mechanism, WC and YH modified and checked the manuscript. All authors read and approved the final manuscript.
